# Schwann cells acquire a repair phenotype after assembling into spheroids and show enhanced in vivo therapeutic potential for promoting peripheral nerve repair

**DOI:** 10.1002/btm2.10635

**Published:** 2023-12-26

**Authors:** Shih‐Heng Chen, Hsin‐Wen Wang, Pei‐Ching Yang, Shih‐Shien Chen, Chia‐Hsin Ho, Pei‐Ching Yang, Ying‐Chi Kao, Shao‐Wen Liu, Han Chiu, Yu‐Jie Lin, Er‐Yuan Chuang, Jen‐Huang Huang, Huang‐Kai Kao, Chieh‐Cheng Huang

**Affiliations:** ^1^ Department of Plastic and Reconstructive Surgery Linkou Chang Gung Memorial Hospital Taoyuan Taiwan; ^2^ School of Medicine College of Medicine, Chang Gung University Taoyuan Taiwan; ^3^ Institute of Biomedical Engineering National Tsing Hua University Hsinchu Taiwan; ^4^ Graduate Institute of Biomedical Materials and Tissue Engineering, College of Biomedical Engineering, International Ph.D. Program in Biomedical Engineering, Taipei Medical University Taipei Taiwan; ^5^ Cell Physiology and Molecular Image Research Center Taipei Medical University–Wan Fang Hospital Taipei Taiwan; ^6^ Department of Chemical Engineering National Tsing Hua University Hsinchu Taiwan

**Keywords:** cell spheroid, cell therapy, peripheral nerve injury, regenerative medicine, Schwann cell

## Abstract

The prognosis for postinjury peripheral nerve regeneration remains suboptimal. Although transplantation of exogenous Schwann cells (SCs) has been considered a promising treatment to promote nerve repair, this strategy has been hampered in practice by the limited availability of SC sources and an insufficient postengraftment cell retention rate. In this study, to address these challenges, SCs were aggregated into spheroids before being delivered to an injured rat sciatic nerve. We found that the three‐dimensional aggregation of SCs induced their acquisition of a repair phenotype, as indicated by enhanced levels of c‐Jun expression/activation and decreased expression of myelin sheath protein. Furthermore, our in vitro results demonstrated the superior potential of the SC spheroid‐derived secretome in promoting neurite outgrowth of dorsal root ganglion neurons, enhancing the proliferation and migration of endogenous SCs, and recruiting macrophages. Moreover, transplantation of SC spheroids into rats after sciatic nerve transection effectively increased the postinjury nerve structure restoration and motor functional recovery rates, demonstrating the therapeutic potential of SC spheroids. In summary, transplantation of preassembled SC spheroids may hold great potential for enhancing the cell delivery efficiency and the resultant therapeutic outcome, thereby improving SC‐based transplantation approaches for promoting peripheral nerve regeneration.

**Translational Impact Statement**

This study addresses the limitations of exogenous Schwann cell (SC) transplantation by using preassembled SC spheroids, which (1) induce the transformation of SCs into a repair phenotype, (2) optimize the efficacy of SC‐derived secretome in facilitating essential cell types for peripheral nerve regeneration, and (3) improve cell delivery efficiency, thus enhancing the overall therapeutic efficacy in promoting sciatic nerve repair in rats. Remarkably, this approach avoids the use of chemicals/biological agents or genetic manipulation, indicating its strong translational potential for postinjury peripheral nerve regeneration.

## 1 INTRODUCTION

Peripheral nerve injuries (PNIs) can impair the sensory, motor and autonomic functions of patients, resulting in long‐term disability that diminishes their quality of life. Although injury to the peripheral nervous system, which is generally considered to exhibit greater healing capacity than the central nervous system, can initiate endogenous reparative mechanisms, regeneration following PNI does not guarantee successful functional restoration. A suboptimal prognosis is mainly attributed to the slow rate of axonal regeneration, which leads to neurite outgrowth of only 1 mm per day,^1^ resulting in an extended period before distal targets can be reinnervated. In turn, a prolonged denervation period may result in target tissue atrophy, significantly impeding functional regeneration.^2^

In addition to that of neuronal cells, successful regeneration of damaged peripheral nerves depends on the action of non‐neuronal cells, particularly Schwann cells (SCs). PNI‐induced axonal breakdown induces the dedifferentiation of myelinated SCs, which acquire an immature phenotype with reparative potential.^3^ By releasing cytokines and chemokines, SCs recruit macrophages, which clear myelin debris. Furthermore, reparative SCs align longitudinally to form bands of Büngner, which guide regenerating axons toward denervated target tissues.^4^ Showing this high potential to shape a regeneration‐promoting microenvironment in nerve stumps, transplantation of exogenous SCs has been shown to be a promising cell‐based strategy for promoting structural repair and functional recovery after PNI.^5^ Nevertheless, SC‐based therapeutic approaches have been hindered by the limited availability of SCs.^6^

In addition to the difficulty in obtaining the large quantities of SCs needed for treatment, the success of cell transplantation‐based therapies depends on the efficacious engraftment of injected cells into target tissues.^7^, ^8^, ^9^ However, typical graft preparation procedures for cell transplantation involve dissociation and suspension of cultured SCs, which may trigger a form of programmed cell death induced by cell detachment known as anoikis.^7^, ^10^ Furthermore, successful transfer of all SCs into a lesion tissue can be technically difficult, as a significant portion of the administered cells may leak from the target area.^7^, ^11^ Although biomaterial‐based strategies that utilize conduits^12^, ^13^ or hydrogels^14^, ^15^ have been proposed to improve the overall SC delivery efficiency, the byproducts generated during polymer degradation may induce local inflammation and can be neurotoxic.^16^, ^17^ Therefore, strategies that can (i) maximize the therapeutic potential of a limited number of SCs and (ii) increase the efficiency of SC delivery to lesion sites and avoid the use of foreign materials are urgently needed to realize the clinical translation of SC‐based therapies.

Recently, the exposure of cells grown in a three‐dimensional (3D) configuration to a milieu that better recapitulates the microenvironments in living tissues than that of cells cultivated in conventional monolayers has been reported.^18^, ^19^, ^20^ As a result, the regenerative effects of cells grown as spheroids can be efficiently upregulated.^18^, ^19^, ^20^ Furthermore, according to experimental results that we previously reported^21^, ^22^, ^23^ and those reported by other groups,^20^, ^24^, ^25^ cells that are transplanted as spheroids show enhanced acute retention and survival rates compared to those injected in single‐cell suspensions.

Although spheroid‐based cell therapies seem to be promising, whether the assembly of SCs into 3D structures can enhance their therapeutic potential for treating PNI has not been investigated to date. In this study, we hypothesized that transplantation of SCs that have been cultivated in a spheroid conformation can address the abovementioned issues and enhance the overall therapeutic benefits by (i) promoting SC functionality and (ii) increasing cell delivery efficiency. Herein, rat SCs were harvested and aggregated into spheroids. SC behaviors in terms of transformation into a repair phenotype, paracrine activity, and modulation of other crucial cell types (neurons, endogenous SCs, and macrophages) involved in peripheral nerve regeneration were explored. Moreover, a rat sciatic nerve transection model was utilized to evaluate the engraftment efficacy of the prepared SC spheroids. Animal gait, compound muscle action potentials (CMAPs), degree of muscle atrophy, and myelin sheath thickness were assessed to verify the in vivo therapeutic potential of SC spheroids.

## 2 RESULTS

### 2.1 The ECM and secretome released from SCs are retained in spheroids

**FIGURE 1:**
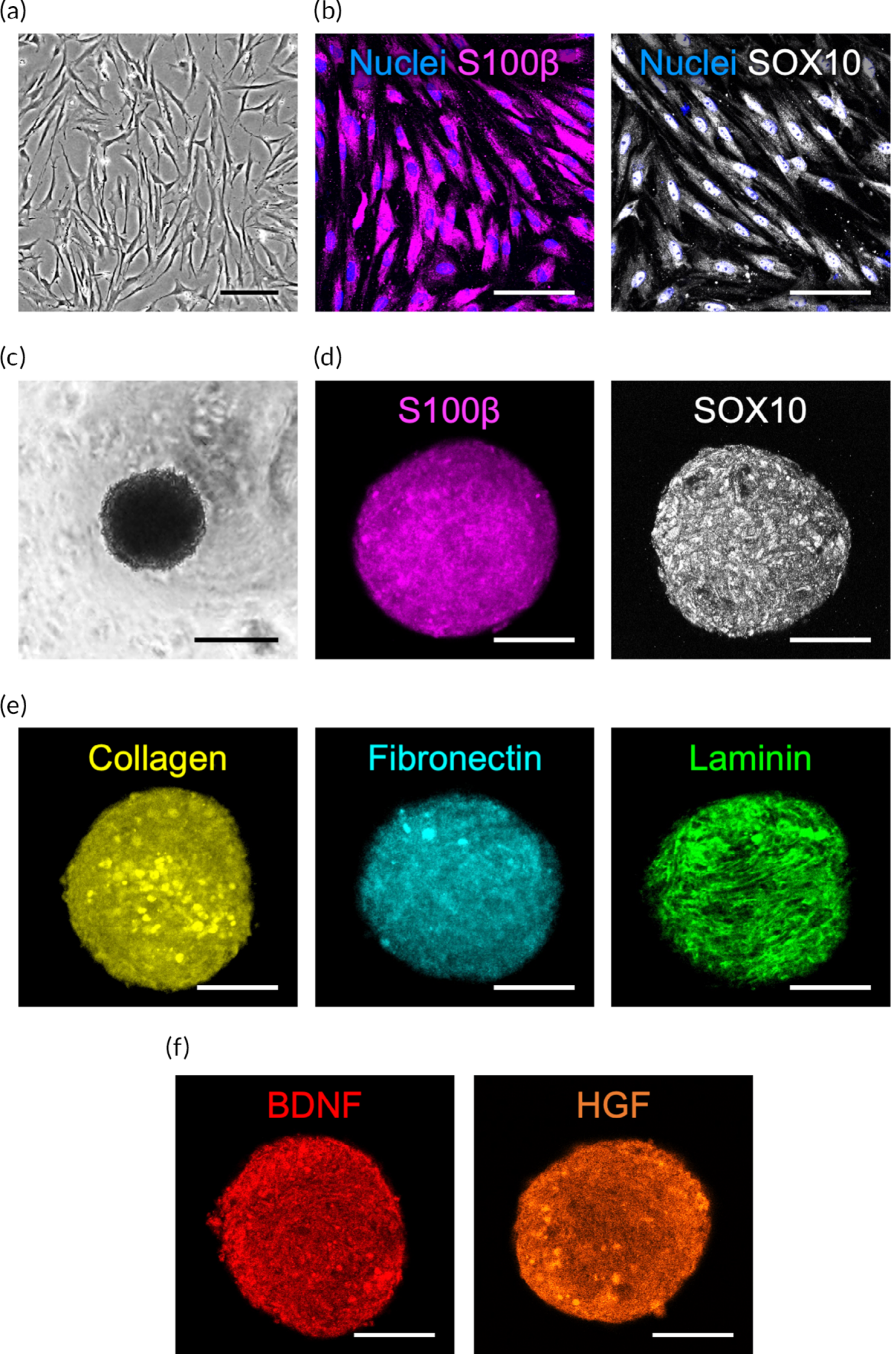
Schwann cell (SC) spheroids contain abundant extracellular matrix (ECM) proteins and paracrine factors. Representative (a) phase‐contrast photomicrograph showing isolated rat SCs and (b) fluorescence images showing their expression of SC markers S100β and SOX10. (c) Representative phase‐contrast photomicrograph showing an assembled SC spheroid. Confocal images showing the expression of (d) S100β and SOX10, (e) ECM, and (f) paracrine factors in SC spheroids. Scale bar, 200 μm in (a and c); 100 μm in (b, d–f).

The SCs isolated from rat sciatic nerves displayed an elongated morphology (Figure 1a), similar to that reported in the literature.^26^ As SCs are known to express a significantly higher level of d‐amino acid oxidase (an enzyme essential for metabolizing d‐valine),^26^ a purity level of >95% was achieved in the obtained SCs using a basal medium containing d‐valine instead of l‐valine. This was evidenced by the results obtained from S100β and SOX10 fluorescence staining (Figure 1b). To determine whether spheroids show greater therapeutic potential than SCs delivered in a single‐cell suspension, we first established SC spheroids in culture. SC spheroids were prepared by using methylcellulose hydrogel‐coated 96‐well plates, which provided a nonattachable surface and facilitated spontaneous spheroid assembly.^21^, ^22^, ^23^, ^27^ In the present study, 10,000 SCs suspended in culture medium were loaded into each well of a plate to facilitate spheroid assembly. After 24 h of cultivation, the SC spheroids had formed with a diameter of 242.3 ± 17.8 μm (Figure 1c) and were ready to be harvested for analysis or transplantation.

The formation of SC spheroids was confirmed by confocal microscopy showing the SC markers S100β and SOX10, as presented in Figure 1d. We next explored whether extracellular matrices (ECMs) and the associated soluble factors were present in the cell spheroids. Fluorescence images revealed that matrix proteins (collagen type I, fibronectin, and laminin; Figure 1e) and growth factors (brain‐derived growth factor [BDNF] and hepatocyte growth factor [HGF]; Figure 1f) were dispersed within the SC spheroids, demonstrating the preservation of the ECM and SC secretome in the collected spheroids.

### 2.2 Assembly into spheroids promotes the conversion of SCs into a repair phenotype

SCs exhibit plasticity and undergo shifts between mature (myelin) and immature (repair) phenotypes.^28^, ^29^ Therefore, following confirmation of SC spheroid formation, we then aimed to investigate whether assembly into spheroids affected the SC phenotype by assessing the activation of c‐Jun, a transcription factor that governs the global response of SCs to injury, and the expression of myelin protein zero (MPZ), a major component in the myelin sheath of peripheral nerves. SCs that were cultivated in monolayers and harvested by trypsinization were used as controls.

**FIGURE 2:**
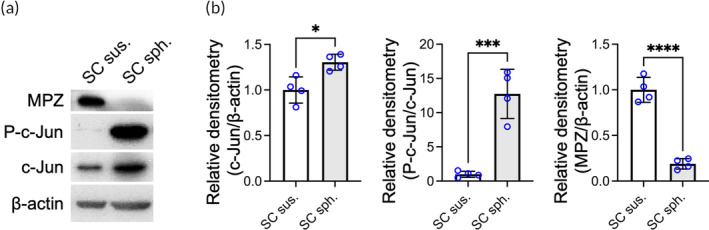
Schwann cells (SCs) assembled as spheroids acquire a repair phenotype. (a) Western blots showing myelin protein zero (MPZ), c‐Jun, phosphorylated c‐Jun (P‐c‐Jun), and β‐actin in SCs in single‐cell suspension (sus.) or spheroid (sph.) configuration, and (b) the corresponding protein levels normalized to the level of β‐actin (*n* = 4). The data are presented as the mean ± SD. All *p* values were calculated by two‐tailed Student's *t* test. *p < .05; ***p < .005; ****p < .001.

According to our immunoblot results (Figure 2a) and corresponding quantitative analysis (Figure 2b), aggregation of SCs into spheroids led to a significant decrease in MPZ protein expression (0.19‐fold that of the SCs in single‐cell suspension; *p* < .001), suggesting the suppression of myelin development. Furthermore, we found that in the SC spheroids, c‐Jun expression was increased (1.3‐fold; *p* < .05), and the level of c‐Jun phosphorylation, suggesting c‐Jun activation, was substantially elevated (12.7‐fold; *p* < .005; Figure 2a,b). Notably, c‐Jun expression and phosphorylation/activation have been reported to be essential for SC reprogramming and to initiate the subsequent repair of neuronal tissue after PNI.^28^, ^30^

The mRNA levels of *Egr1* (a marker for nonmyelinating SCs)^31^ and *Egr2* (a marker for myelinated SCs)^31^ in SCs collected as single‐cell suspensions and spheroids were further determined using real‐time quantitative polymerase chain reaction (qPCR). Although the mRNA level of *Egr2* was comparable between the two investigated groups (*p* > .05), a significant 2.5‐fold upregulation of *Egr1* (*p* < .005) was observed in SC spheroids compared to SC suspensions (Figure S1), suggesting negative regulation of myelination in SCs grown as spheroids. Therefore, our experimental results obtained from Western blotting and qPCR demonstrated that SCs assembled as spheroids acquired a reparative phenotype compared to those in the single‐cell suspension.

### 2.3 Assembly of SCs into a 3D spheroid configuration enhances their paracrine activity

One of the critical functions of c‐Jun in SCs is the modulation of paracrine signaling to support the survival and regeneration of neurons after PNI.^32^ Since increased c‐Jun expression and phosphorylation were observed in SC spheroids, we next sought to explore the expression of paracrine factors in SCs. Therefore, SCs harvested as single‐cell suspensions or spheroids were seeded in culture plates and cultivated for 48 h prior to gene expression analysis. Moreover, the generated conditioned medium (CM) was collected and analyzed using a rat cytokine antibody array.

**FIGURE 3:**
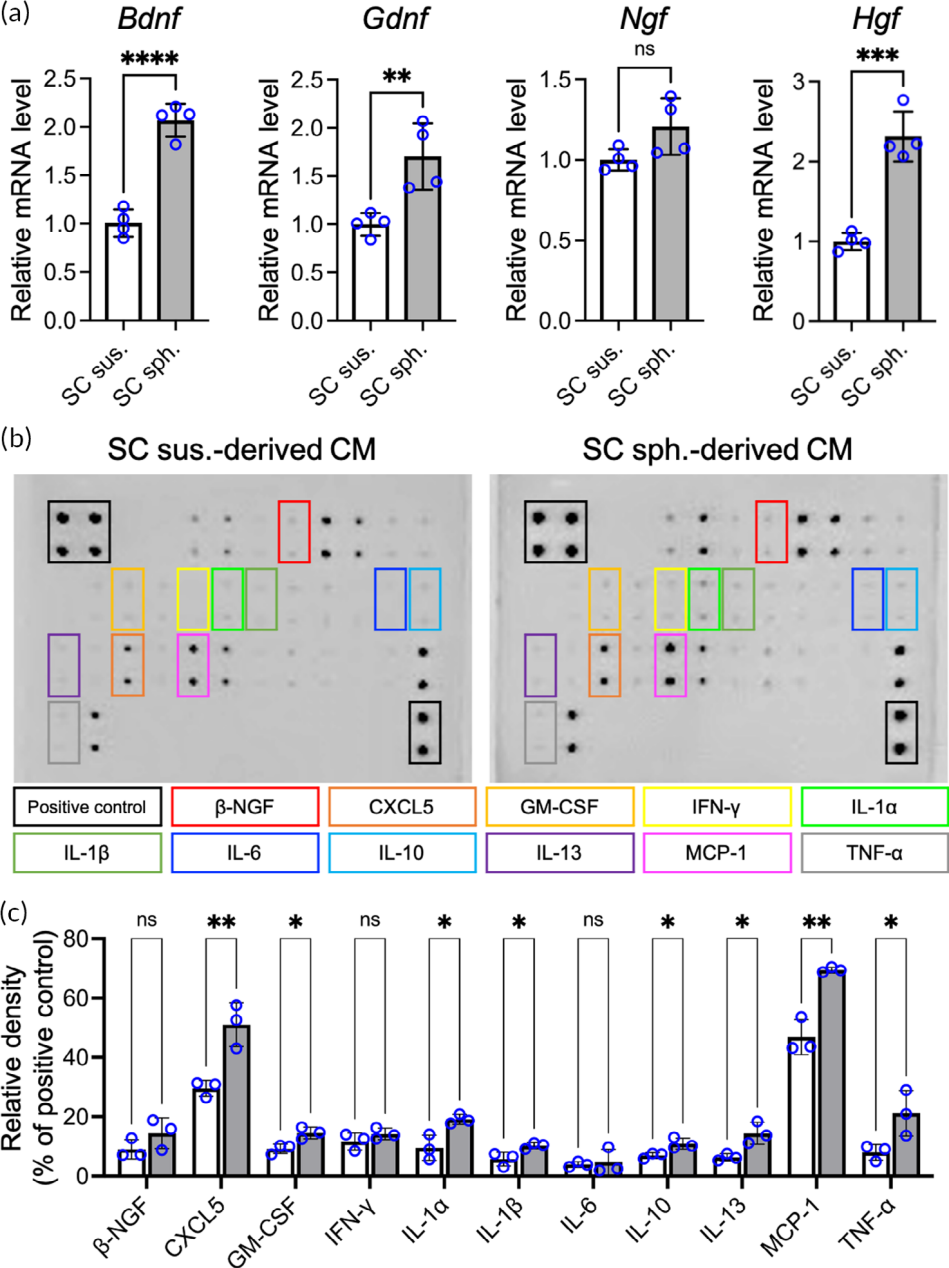
Schwann cells (SCs) assembled as spheroids show enhanced paracrine potential. (a) Relative mRNA levels of neurotrophic factors in SCs in a single‐cell suspension or spheroid configuration. (*n* = 4). (b) Representative results of cytokine antibody array analysis and (c) corresponding signal intensity normalized to the positive control. (*n* = 3). Data are presented as the mean ± SD. All *p* values were calculated using two‐tailed Student's *t* test. *p < .05; **p < .01; ns, not significant.

According to the results of qPCR, significant upregulation of *Bdnf* (2.07‐fold; *p* < .001), *Gdnf* (1.71‐fold; *p* < .01), and *Hgf* (2.31‐fold; *p* < .005) was observed in SC spheroids compared to cells seeded as a single‐cell suspension (Figure 3a); however, the mRNA level of *Ngf* was comparable between the two investigated groups (*p* > .05). Furthermore, an antibody array assay confirmed the increase in the levels of 11 cytokines in the CM derived from SC spheroids compared to that derived from SC suspensions (*p* < .05; Figure 3b,c). Among the cytokines with increased levels, C‐X‐C motif chemokine 5 (CXCL5; 1.73‐fold; *p* < .01), granulocyte macrophage colony‐stimulating factor (GM‐CSF; 1.58‐fold; *p* < .05), interleukin (IL)‐1α (2.01‐fold; *p* < .05), IL‐1β (1.79‐fold; *p* < .05), IL‐10 (1.57‐fold; *p* < .05), IL‐13 (2.28‐fold; *p* < 0.05), monocyte chemoattractant protein‐1 (MCP‐1; 1.48‐fold; *p* < .01), and tumor necrosis factor (TNF)‐α (2.65‐fold; *p* < .05) were identified. The enhanced expression of neurotrophic factors in SC spheroids and the enriched cytokine content in the SC spheroid‐derived CM suggested that SCs assembled into spheroids may promote SC paracrine activity and increase the potential of SCs to support regeneration and modulate inflammation in injured peripheral nerves.

### 2.4 The secretome of SC spheroids promotes the neurite outgrowth of dorsal root ganglion neurons

As the aggregation of SCs into spheroids efficiently enhanced the mRNA expression of neurotrophic factors, we next investigated whether SC spheroids promote the axonogenesis of neurons to a greater extent than SCs in suspension. To this end, dorsal root ganglion (DRG) neurons isolated from adult rats were treated with CM from SC suspensions or spheroids. Neurons cultured with basal medium constituted the control group.

**FIGURE 4:**
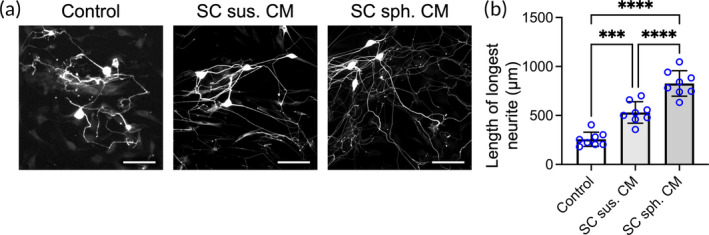
The secretome of Schwann cell (SC) spheroids promotes neurite outgrowth of dorsal root ganglion (DRG) neurons. (a) Fluorescence images showing stained βIII tubulin in DRG neurons cultured in SC‐derived conditioned medium (CM). Scale bar, 100 μm. (b) The longest neurite lengths measured among DRG neurons (*n* = 8). The data are presented as the mean ± SD. All *p* values were calculated by one‐way ANOVA with Tukey's correction. ***p < .005; ****p < .001.

After a 48‐h incubation, the formed neurites were measured after immunostaining for βIII tubulin. As revealed by the fluorescence images (Figure 4a) and the corresponding quantitative results indicating the longest neurite (Figure 4b), DRG neurons treated with SC suspension‐derived CM exhibited increased neurite outgrowth compared to that of the untreated control (with an average length of the longest neurite of 530 ± 110 and 257 ± 73 μm, respectively; *p* < .005). However, longer neurites were observed in the SC spheroid‐derived CM‐treated group (827 ± 129 μm; *p* < .001 vs. control or SC suspension‐derived CM). These results indicated the superior efficacy of SC spheroids in enhancing neuron axonogenesis mediated via secretome‐mediated interactions.

### 2.5 The secretome of SC spheroids promotes the proliferation and migration of neighboring SCs

In addition to inducing axonogenesis, we next sought to investigate the modulatory effects of exogenously engrafted SC spheroids on endogenous SCs in peripheral nerves using in vitro models. For this purpose, CM derived from SC suspensions or spheroids (exogenous SCs) was collected and utilized to treat SCs grown in culture plates (endogenous SCs). Cell proliferation assays and wound healing assays were performed to assess whether the secretome of the transplanted SC spheroids promotes the proliferation and migration of endogenous SCs, which are indispensable cellular processes during peripheral nerve regeneration.^29^

**FIGURE 5:**
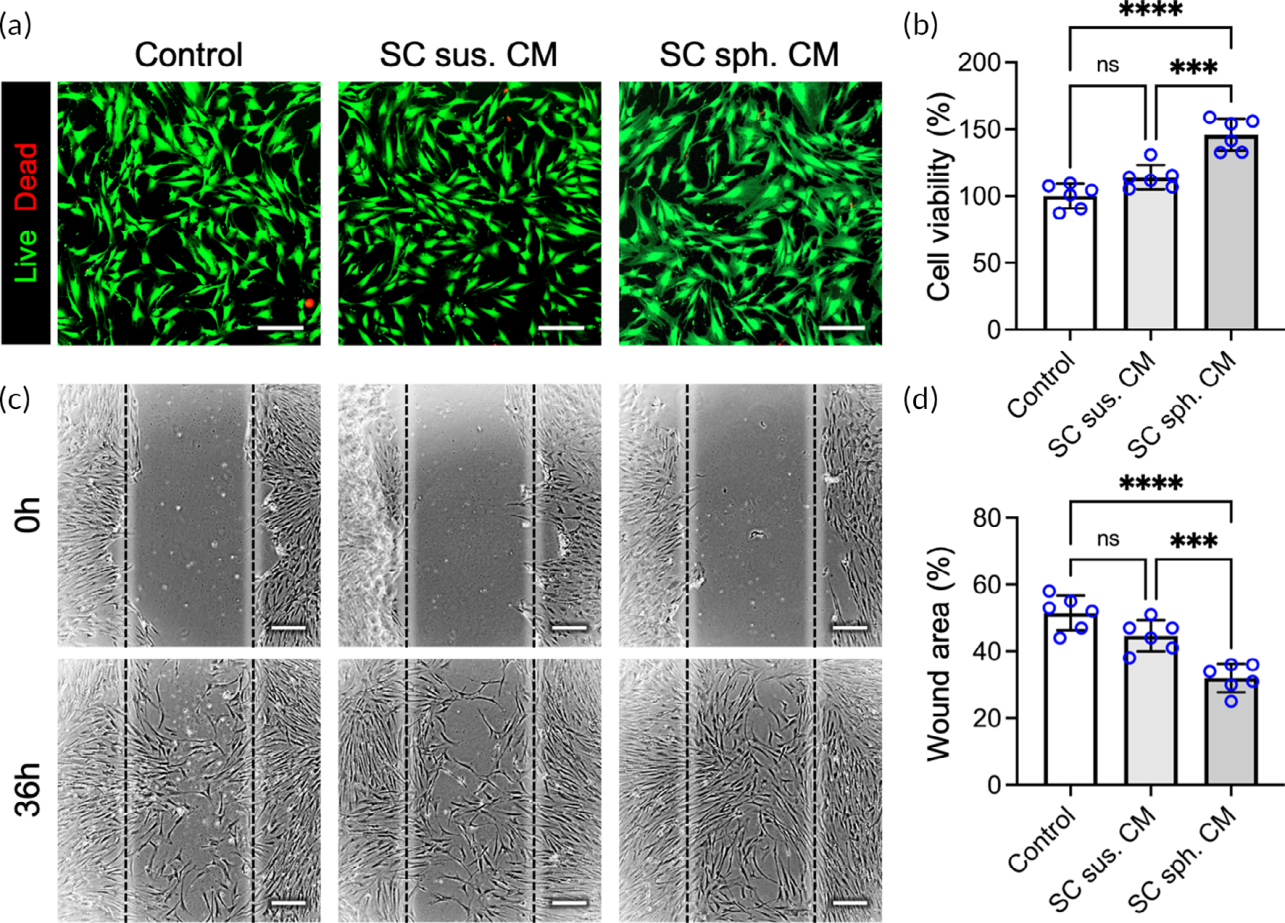
The secretome of Schwann cell (SC) spheroids enhances SC proliferation and migration. (a) Representative images showing SCs treated with SC‐derived conditioned medium (CM) after live/dead staining and (b) the corresponding results of a Cell Counting Kit‐8 assay (*n* = 6). Scale bars, 100 μm. (c) Representative images of SCs cultured in SC‐derived CM in a wound healing assay and (d) the corresponding area of the wound region (*n* = 6). Scale bars, 200 μm. The data are presented as the mean ± SD. All *p* values were calculated by one‐way ANOVA with Tukey's correction. ***p < .005; ****p < .001; ns, not significant.

As indicated by the fluorescence images of live/dead staining (Figure 5a) and the results of a Cell Counting Kit‐8 assay (Figure 5b), SC suspension‐derived CM failed to stimulate SC proliferation (*p* = .076). Nevertheless, a significant increase in the number of cells was observed in the SC spheroid‐derived CM‐treated group (45.8 ± 11.8%; *p* < .001 vs. the control group; *p* < .005 vs. the SC suspension CM group), suggesting exceptional potential of the SC spheroid‐derived secretome to promote SC proliferation.

In the in vitro wound healing assay, CM derived from SC suspensions or spheroids was utilized to treat scratched SC monolayers. As revealed in the photomicrographs (Figure 5c) and the results of the gap area quantification data (Figure 5d), significantly more cells were observed in the wound region in the group treated with SC spheroid CM (31.5 ± 5.2% of the original gap area) than in the control (51.5 ± 5.1%; *p* < .001) or SC suspension CM‐treated group (44.7 ± 4.7%; *p* < .005) after 48 h in culture. The aforementioned experimental results demonstrated that the secretome of exogenously delivered SC spheroids shows great potential for modulating the proliferation and migration of endogenous SCs, thereby benefiting the overall efficacy of peripheral nerve regeneration.

### 2.6 The secretome of SC spheroids recruits macrophages

In addition to neurons and SCs, the presence of macrophages at the injured site of peripheral nerves and their interaction with SCs are reported to be critical for nerve repair.^33^, ^34^ Hence, we then assessed the effect of the SC spheroid‐derived secretome on macrophages. Herein, a Transwell migration assay was conducted by seeding RAW 264.7 macrophages in the inserts and supplying controlled medium or CM derived from SC suspensions or spheroids in the plates. After 24 h of incubation, the cells that migrated across the membrane were detected by crystal violet staining.^33^

**FIGURE 6:**
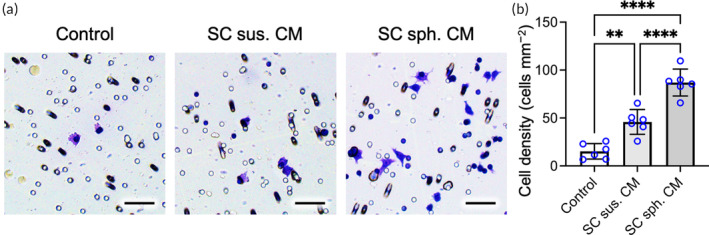
The secretome of Schwann cell (SC) spheroids recruits macrophages. (a) Representative images of the Transwell migration assay showing macrophage translocation across the membrane in response to SC‐derived conditioned medium (CM) and (b) the corresponding density of migrated cells (*n* = 6). Scale bars, 100 μm. The data are presented as the mean ± SD. All *p* values were calculated by one‐way ANOVA with Tukey's correction. **p < .01; ****p < .001.

As indicated by the photomicrographs (Figure 6a) and the corresponding cell density (Figure 6b), more macrophages were identified on the lower surface of the insert membrane in the SC spheroid‐derived CM group (86.9 ± 14.1 cells mm^−2^) than in the SC suspension‐derived CM group (45.8 ± 12.9 cells mm^−2^; *p* < .001) or control group (15.2 ± 8.1 cells mm^−2^; *p* < .001), suggesting the superior capacity of the SC spheroid‐derived secretome in recruiting macrophages.

### 2.7 Transplantation of SCs as spheroids into sutured rat sciatic nerve stumps increases SC retention and engraftment rate

To evaluate the therapeutic efficacy of SC spheroids for treating PNI, a sciatic nerve transection/repair model was surgically established with adult SD rats. Immediately after transection, the traumatized nerve was reconstructed by direct end‐to‐end coaptation, and SC spheroids were administered to both ends of the nerve stump. Animals that received equal amounts of SCs in single‐cell suspension or normal saline were utilized as control rats. To monitor the distribution of the delivered cells, SCs were labeled with the lipophilic dye CM‐DiI prior to transplantation. The treated sciatic nerves were harvested at 7 days postoperation. Additional fluorescent dye was added to stain the plasma membrane and nucleus, and the tissues were optically cleared in preparation for high‐resolution 3D confocal imaging.^35^, ^36^

**FIGURE 7:**
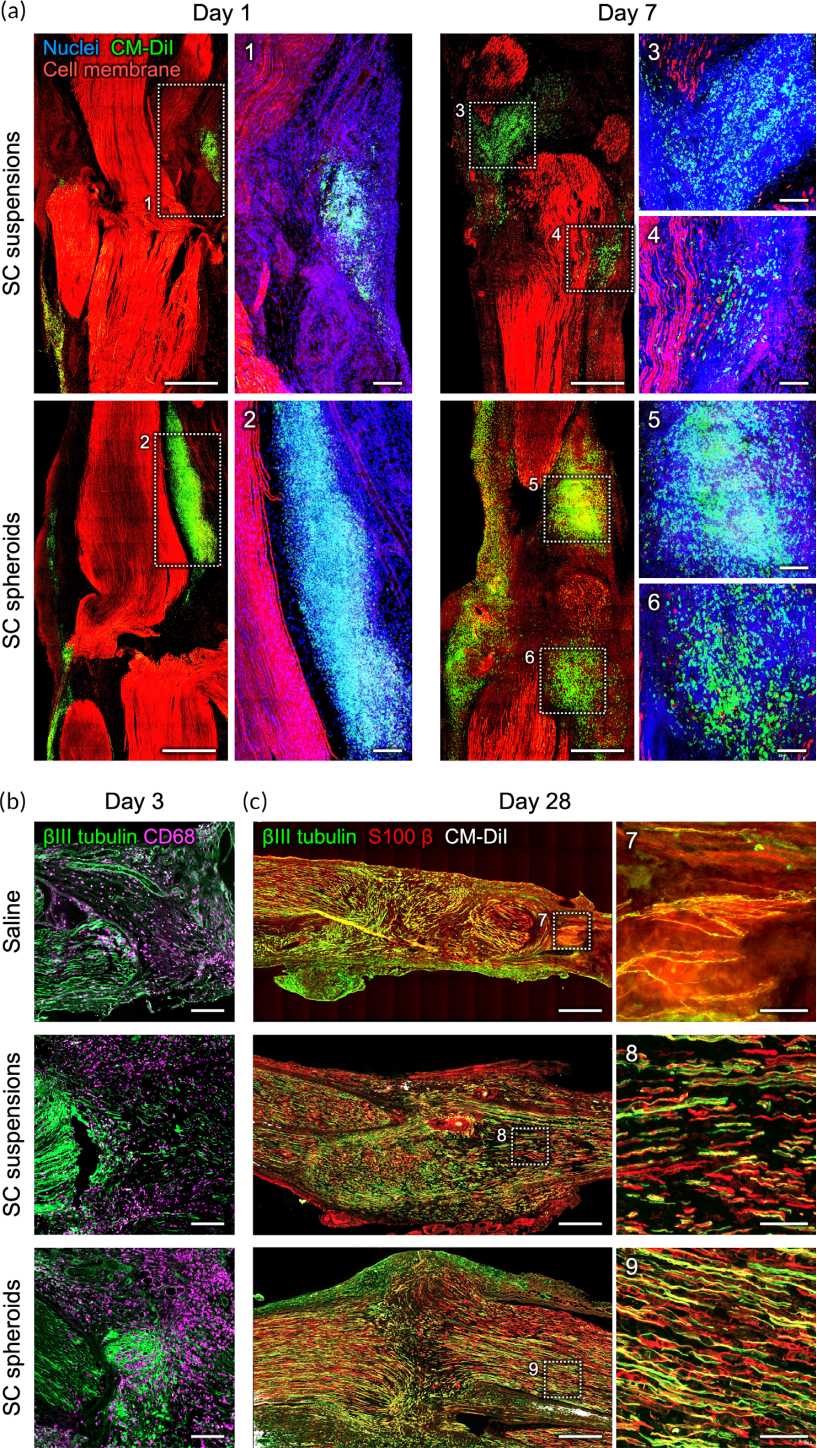
Transplantation of Schwann cells (SCs) preassembled as spheroids shows improved cell retention. (a) Representative confocal images of sutured nerve stumps at 1 or 7 days after transection and administration of conditioned medium (CM)‐DiI‐labeled SCs in a single‐cell suspension or spheroid configuration. Scale bars, 1 mm. Scale bars in insert, 200 μm. Confocal images showing the presence of (b) CD68‐positive macrophages and (c) βIII tubulin‐positive neurites and S100β‐positive SCs in the repaired nerve. Scale bars in (b), 200 μm. Scale bars in (c), 500 μm. Scale bars in (c) insert, 100 μm.

One day after treatment, more CM‐DiI‐labeled cells were detected in the group that received SC spheroids than in the group that received SC suspensions (Figure 7a), suggesting that the acute retention rate of injected cells could be improved when cells were delivered as 3D spheroids. Seven days after cell administration, only a limited number of CM‐DiI‐positive cells were detected in the retrieved sciatic nerves treated with SC suspensions, revealing significant cell loss (Figure 7a and Supplementary Video 1). In animals treated with SC spheroids, however, a substantial number of engrafted cells were observed within the sciatic nerve (Figure 7a and Supplementary Video 2), demonstrating the enhanced acute cell retention rate and cell delivery efficiency of the cell spheroid‐based transplantation approach for the sciatic nerve.

We then further monitored the presence of macrophages in the injured sciatic nerve. As indicated by our confocal images in Figure 7b, more CD68‐positive cells were detected in the nerves treated with SC spheroids than in those treated with SC suspensions or saline, suggesting an enhanced macrophage recruitment potential of SCs when assembled as spheroids. Furthermore, at 4 weeks postoperatively, we observed the extension of neurites across the injury site in all three investigated groups (Figure 7c). However, importantly, the density of neurites and their interaction with SCs were significantly greater in the SC spheroid‐treated group than in the control groups, signifying a substantial enhancement in peripheral nerve repair.

### 2.8 Grafting of SC spheroids promotes rat locomotor functional recovery by attenuating muscle atrophy and promoting axon myelination after PNI

**FIGURE 8:**
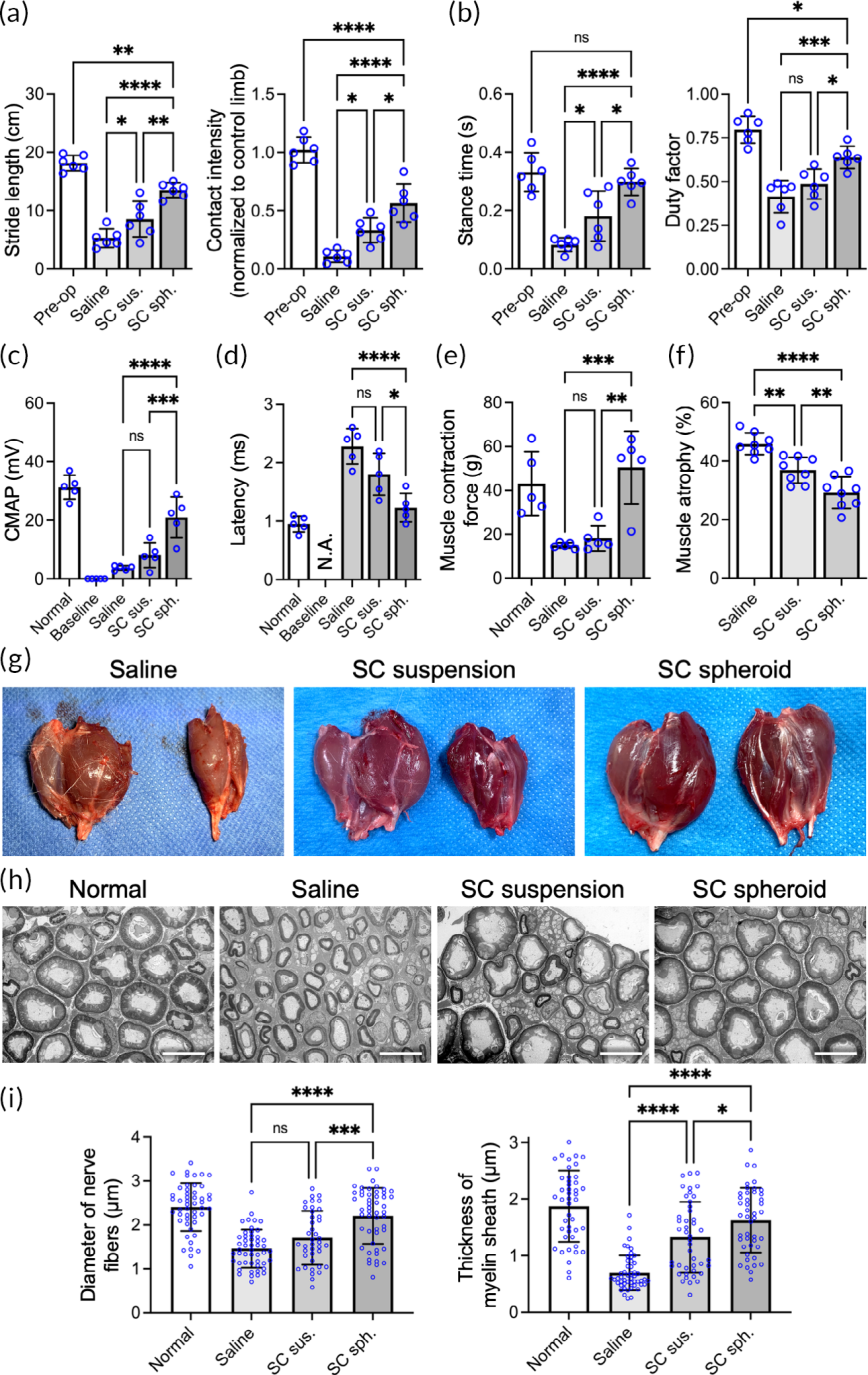
Transplantation of Schwann cell (SC) spheroids promotes the structural and functional repair of injured peripheral nerves. Three months after injury, the (a) spatial and (b) temporal patterns of gait (*n* = 6), (c) compound muscle action potential (*n* = 5), (d) latency (*n* = 5), and (e) isometric muscle contraction force (*n* = 5) were analyzed. (F, G) The gastrocnemius muscle was harvested to evaluate the extent of muscle atrophy (*n* = 8). (h) Representative transmission electron microscopy images of the sciatic nerve distal to the injury site. Scale bar, 10 μm. (i) Diameter of the nerve fiber and thickness of the myelin sheath (*n* = 5 animals in each group; 10 images were analyzed from each animal). The data are presented as the mean ± SD. All *p* values were calculated by one‐way ANOVA with Tukey's correction. *p < .05; **p < .01; ***p < .005; ****p < .001; ns, not significant.

A gait analysis was conducted to monitor rat locomotor function. Three months postoperation, SC transplantation effectively accelerated the motor function recovery of the rats that underwent sciatic nerve transection/repair, as indicated by the spatial (Figure 8a) and temporal (Figure 8b) patterns of their gaits. Specifically, animals treated with SC spheroids exhibited a remarkable increase in stride length (*p* < .01) and paw contact intensity (*p* < .05) compared to those treated with SC suspensions (Figure 8a). Furthermore, greater therapeutic efficacy was observed in the SC spheroid group than in the SC suspension group in terms of limb stance time (*p* < .05) and duty factor (defined as the ratio of stance time to stride time; *p* < .05; Figure 8b).

Next, CMAPs and latency were recorded from the gastrocnemius muscle to evaluate the degree of reinnervation. The CMAP amplitude in SC spheroid‐treated animals was significantly higher than that in animals treated with saline or SC suspension (*p* < .01 and *p* < .05, respectively; Figure 8c). Furthermore, a shorter latency was observed in SC spheroid‐treated animals than in the other groups, indicating better nerve conductivity (Figure 8d). Moreover, the maximal isometric contraction force exerted by the gastrocnemius and soleus muscles was significantly increased after SC spheroid transplantation (*p* < .005 vs. saline; *p* < .01 vs. SC suspension; Figure 8e). Collectively, these results suggest that the engraftment of SCs as spheroids induced superior therapeutic efficacy by increasing the electrophysiological function of limbs after peripheral nerve repair.

The gastrocnemius muscles of the experimental animals were harvested to assess the degree of atrophy, which is defined as the percentage of the decreased muscle weight of an affected limb to that of the contralateral healthy limb.^37^ While injection of SC suspensions ameliorated PNI‐induced muscle weight loss (36.9 ± 4.4% vs. 45.8 ± 3.7% in saline group; *p* < .01), transplantation of SC spheroids was more effective in attenuating muscle atrophy (29.2 ± 5.3%; *p* < .001 vs. saline group; *p* < .01 vs. SC suspension group; Figure 8f,g). Finally, the harvested sciatic nerves were examined by transmission electron microscopy (TEM). As revealed by the representative TEM images (Figure 8h) and the corresponding quantitative results (Figure 8i), nerve fibers with larger diameters and thicker myelin sheaths were observed in the SC spheroid group than in the saline group (*p* < .001) or the SC suspension group (*p* < .05). The aforementioned experimental data provide solid evidence showing the high therapeutic potential of SCs assembled as spheroids for treating PNI.

## 3 DISCUSSION

SC transplantation‐based therapies have been considered promising approaches to accelerate the repair of injury to the peripheral nervous system^5^, ^6^, ^38^, ^39^ and even to the central nervous system.^7^, ^40^ Despite the aforementioned superiority of the translation potential of SC transplantation, procedures to optimize therapeutic efficacy and delivery efficiency have not been established to date, hampering SC clinical applications. In the present study, major challenges to SC‐based therapeutic approaches were addressed via transplantation of SCs that preassembled into spheroids. Herein, we successfully demonstrated that (i) SCs assembled into spheroids acquired a repair phenotype and showed enhanced potential to promote neuron axonogenesis, modulate endogenous SC proliferation and migration, and recruit macrophages, and (ii) SCs transplanted as 3D spheroids showed increased cell delivery efficiency compared to SCs injected in conventional single‐cell suspension.

To induce the clustering and aggregation of cells, SCs were introduced into the culture wells with a methylcellulose‐coated surface, a method that has been reported for the efficient preparation of spheroids using stem/non‐stem cells by our group and other groups.^21^, ^22^, ^23^, ^27^, ^41^, ^42^ While a hypoxic core is typically expected to form within the interior of cell spheroids, we posited that the likelihood of necrotic core development was reduced due to the relatively brief spheroid culture duration (<24 h) and the fact that the assembled spheroids had a radius of approximately 120 μm, which remained within the range for oxygen diffusion.^43^, ^44^

In the harvested cell spheroids, abundant ECM proteins deposited by SCs were detected, which was in line with our previous investigations in which we used various cell types for spheroid fabrication,^21^, ^22^, ^23^, ^45^ suggesting that a cell–ECM interaction could be maintained throughout the cell‐handling procedures, such collection, quality evaluation and transportation, prior to transplantation. In contrast, SCs that were harvested in a conventional single‐cell suspension via enzymatic dissociation were detached from ECM and neighboring cells after collection, thus rendering them vulnerable to anoikis.^10^, ^46^

In addition to matrix proteins, we observed the presence of BDNF and HGF in SC spheroids. Since soluble factors are bound and sequestered by the ECM,^47^ it was not surprising that the preservation of the ECM led to retention of the soluble factors that had been released by SCs. BDNF has been demonstrated to be capable of suppressing SC anoikis,^10^ whereas HGF is widely recognized for its pro‐survival potential.^48^ Together with other deposited growth/neurotrophic molecules in spheroids, these factors were retained by SCs during cell handling and transplantation, benefiting postengraftment cell survival and functionality.

In addition to identifying an increase in the expression of BDNF and HGF, we found enhanced expression/activation of c‐Jun in the fabricated SC spheroids. The response of adult SCs to PNI is a c‐Jun‐dependent reprogramming process.^28^ As a master regulator that controls SC plasticity, the transcription factor c‐Jun modulates the critical reparative activity of SCs, including maintenance of neuron viability, clearance of myelin debris, and support of axon elongation.^28^, ^30^, ^32^, ^49^ Therefore, in the present study, the observed upregulation/activation of c‐Jun‐mediated signaling with a simultaneous decrease in the levels of myelin‐related proteins (e.g., MPZ), which are considered signs of SC phenotypic transition,^50^, ^51^ provides direct evidence that SCs that assembled into spheroids acquired a repair phenotype.

To the best of our knowledge, the spheroid configuration‐induced phenotypic reprogramming of SCs has not been previously reported. Although we show that SC spheroids acquired a reparative phenotype, the overall molecular mechanism underlying this transition has not been clarified and remains to be elucidated. One of the potential mechanisms involves the regulation of SC reprogramming mediated by ECM and soluble factors. SCs have been reported to communicate with each other through matrix proteins.^28^ Furthermore, the laminin and fibronectin that have been previously identified in SC spheroids can modulate multiple SC behaviors via integrin‐mediated signaling^52^ and are essential for promoting axonal regrowth and peripheral nerve regeneration by SCs.^53^, ^54^, ^55^ Moreover, HGF, which was detected in the SC spheroids in this study, has been reported to enhance the reparative potential of SCs by promoting proliferation/migration, neurotrophic factor (such as glial cell line‐derived neurotrophic factor [GDNF]) secretion, and macrophage recruitment.^56^ Therefore, these matrix and soluble factors expressed by SCs in spheroids may shape a microenvironment that promotes SC repair program activation.

As the expression of a variety of neurotrophic factors is directly/indirectly regulated by c‐Jun,^28^, ^32^ the significant upregulation of *Bdnf*, *Gdnf*, and *Hgf* in SC spheroids exhibiting enhanced c‐Jun activation was consistent with our expectation. The enhanced mRNA expression of these neurotrophic factors may have contributed to the increased potential of the SC spheroid‐derived secretome in promoting the neurite outgrowth of DRG neurons. SCs support axonal regeneration through both direct cell contact‐dependent and contact‐independent (secretome‐based) mechanisms.^57^ Although we have successfully demonstrated the superior potential of the SC spheroid‐derived secretome compared to that of SCs in single‐cell suspension, more investigations are required to assess whether the assembly of SCs into spheroids increases their pro‐axonogenic effect via contact‐dependent mechanisms.

Increasing evidence has indicated that neurotrophic factors can modulate the behaviors of SCs in addition to neuronal cells.^58^, ^59^, ^60^ Therefore, the enhanced proliferation and migration of SCs treated with the SC spheroid‐derived secretome may be partially attributed to the enriched content of neurotrophic factors. Our in vitro results suggested that exogenously administered SC spheroids may hold great potential to promote the proliferation and migration of endogenous SCs, which is commonly considered to benefit the efficacy of peripheral nerve regeneration.^61^ SC migration has been reported to direct axon sprouting and is therefore thought to be crucial for nerve repair.^62^ Furthermore, enhanced SC migration has been shown to be associated with accelerated axon regeneration.^62^ The importance of SC proliferation in the repair of PNI, however, has recently been considered to be less significant.^30^, ^56^, ^63^ Further studies are needed to elucidate how engrafted SCs harness endogenous SCs to promote peripheral nerve regeneration.

Successful regeneration of injured peripheral nerves depends on the participation of macrophages and their close interaction with SCs, as macrophages can remove debris to initiate the subsequent reparative process.^34^, ^64^ According to our results of cytokine array analysis, SC spheroids released numerous factors (CXCL5, GM‐CSF, MCP‐1, TNF‐α, and ILs) that can promote macrophage homing and activation at higher levels than SC suspensions. Our results of the Transwell migration assay further confirmed the enhanced macrophage recruitment potential of SC spheroid‐derived CM. Although it has been suggested that enhanced macrophage homing after PNI can lead to efficient debris clearance,^65^ more investigations are required to verify whether the enhanced macrophage recruitment potential of SC spheroids correlates with the observed in vivo therapeutic outcomes.

Transplantation of SCs that had been prepared in conventional single‐cell suspension into injured sciatic nerves led to only marginal improvement in gait function and degree of muscle atrophy; the muscular electrophysiological performance and the diameter of distal nerve fibers did not significantly differ in the rats treated with SC suspensions and saline. The suboptimal therapeutic efficacy observed in the SC suspension‐treated group presumably stemmed from the limited quantity and quality of the engrafted SCs.^7^ In contrast, administration of SCs in a 3D spheroid form, which showed enhanced therapeutic capabilities, exhibited a greater retention rate in target tissues and significantly better locomotor functional recovery, nerve reinnervation, and remyelination compared to animals receiving saline or SC suspensions. Our experimental results confirmed the effectiveness of SC spheroid transplantation‐based regenerative therapies for accelerating peripheral nerve repair.

With our approach, patients who suffer from PNI can be treated with 3D spheroids of primary SCs (such as sural nerves^38^, ^66^) or stem cell/non‐stem cell‐derived SCs (pluripotent stem cells,^67^ mesenchymal stem cells,^23^ adipose‐derived stem cells,^68^ or fibroblasts^5^, ^6^) from autologous or allogenic sources. While our proof‐of‐concept study has effectively showcased the heightened therapeutic promise of SC spheroids in the treatment of PNIs, there are still several constraints that must be carefully addressed prior to advancing toward clinical application. First, only female rats were included in the present study. Therefore, further investigations are necessary to determine whether the observed therapeutic effects are associated with gender bias. Second, it has been reported that utilizing allogeneic SCs, which express major histocompatibility complex class I, in peripheral nerve repair may trigger an immune response from the host.^69^, ^70^ The impact of assembling SCs into a spheroid configuration on their immunogenicity remains uncertain and requires investigation to support future clinical translation. Third, the size of the SC spheroids prepared in the present study was not optimized. It is well established that spheroid sizes are closely linked to the microenvironments within the spheroid interior, influencing cellular behaviors and thus therapeutic potential.^41^, ^71^, ^72^, ^73^ Therefore, further research aimed at determining the optimal size of SC spheroids should be conducted before their broader application. Fourth, the phenotype of both the engrafted and endogenous SCs throughout the nerve repair process was not monitored. To gain a deeper understanding, further investigations are essential to determine whether the engrafted SC spheroids sustain a reparative phenotype, the duration of this maintenance, and the point at which they commence differentiation into myelinated SCs. Of paramount importance is elucidating whether the introduction of exogenous SC spheroids synergizes and integrates with endogenous SCs to expedite the overall reparative progress, a crucial aspect that remains currently unknown. Fifth, the present study employed SCs derived from rats. Further investigations employing human‐derived SCs are warranted to validate the observed beneficial effects of spheroid assembly with rat SCs before considering translational applications. Finally, a follow‐up study should be performed to evaluate the deleterious effect because of the injection volume, and the repaired nerve should be monitored to assess the risk of nerve scarring induced by cell administration.

## 4 CONCLUSION

In conclusion, this research study demonstrated that the assembly of SCs into 3D spheroids enhanced the paracrine activity of the cells and promoted the acquisition of a repair phenotype, potentially increasing the capacity of SCs to support regeneration in injured peripheral nerves. Overall, the findings of this study suggest that SC spheroids may be a promising approach to develop more effective SC‐based therapies for PNI.

## 5 MATERIALS AND METHODS

### 5.1 Animals

All animal protocols were approved by the Institutional Animal Care and Utilization Committee of National Tsing Hua University (IACUC Approval No. 10641) and Chang Gung Memorial Hospital (IACUC Approval No. 2022071201). All procedures were conducted in accordance with the Guideline for the Care and Use of Laboratory Animals issued by the Council of Agriculture, Executive Yuan, Taiwan in 2018 and in compliance with the ARRIVE guidelines. Six‐week‐old female Sprague–Dawley (SD) rats were obtained from BioLASCO (Taipei, Taiwan) for SC extraction and sciatic nerve transection. Embryonic SD rats from pregnant females at 15 days gestation were also sourced from BioLASCO and used for DRG neuron isolation.

### 5.2 Cell culture

Primary SCs were isolated according to a protocol established by Kaewkhaw et al.^26^, ^37^ Briefly, sciatic nerves excised from rats were first placed into ice‐cold Dulbecco's modified Eagle's medium (DMEM; Thermo Fisher Scientific, Waltham, MA, USA) with 100 units ml^−1^ penicillin, 100 μg ml^−1^ streptomycin, and 250 ng ml^−1^ amphotericin B (GeneDireX; Taipei, Taiwan). After stripping off the connective tissues, the nerve fragments were digested with 0.05% collagenase I (w v^−1^; Sigma‐Aldrich, St. Louis, MO, USA) for 60 min at 37°C. The acquired cells were cultured in Minimum Essential Medium Eagle d‐valine (United States Biological; Salem, MA, USA) supplemented with 10% fetal bovine serum (FBS; Corning, Corning, NY, USA), 5 μM forskolin (Sigma‐Aldrich), 1% N2 Supplement, 20 μg ml^−1^ bovine pituitary extract (both from Thermo Fisher Scientific), 100 units ml^−1^ penicillin, 100 μg ml^−1^ streptomycin, and 250 ng ml^−1^ amphotericin B in dishes coated with poly‐l‐lysine and laminin (both from Sigma‐Aldrich). After 20 days, the identification of the isolated SCs was verified by immunostaining against S100β and SOX10.^31^ For subsequent SC expansion, cells were subcultured at a split ratio of 1:3 on poly‐l‐lysine‐coated plates. SCs obtained between passages 4 and 7 were utilized for all experiments. For fluorescence labeling, SCs were treated with Vybrant CM‐DiI Cell‐Labeling Solution (Thermo Fisher Scientific) according to the manufacturer's manual.

To isolate DRG neurons, the ganglia were harvested from embryonic rats and digested with 0.125% trypsin in Leibovitz's L‐15 medium (GeneDireX) for 15 min at 37°C.^74^ Next, the acquired cells were triturated in L‐15 medium supplemented with 5% horse serum (Thermo Fisher Scientific) and 10 mg ml^−1^ DNase I (Sigma‐Aldrich). Finally, the DRG neurons were suspended in neurobasal medium containing 1X B‐27, 100 ng ml^−1^ nerve growth factor, 2 mM GlutaMAX, 100 units ml^−1^ penicillin, and 100 μg ml^−1^ streptomycin (Thermo Fisher Scientific) and plated into Matrigel‐coated μ‐Slide 8 Wells (ibidi; Munich, Germany) at a density of 1500 cells per well.

RAW 264.7 cells were purchased from the Bioresource Collection and Research Center, Food Industry Research and Development Institute (Hsinchu, Taiwan) and maintained in DMEM supplemented with 4 mM glutamine and 10% FBS.

### 5.3 Preparation and characterization of SC spheroids

Cell spheroids were prepared by using a methylcellulose hydrogel‐based method.^21^, ^22^, ^23^, ^27^ Briefly, 12% (w v^−1^) methylcellulose solution was prepared by dissolving methylcellulose powder (Sigma‐Aldrich) in 0.5X phosphate‐buffered saline (Thermo Fisher Scientific), sterilized by autoclaving, and added to 96‐well culture plates (50 μl well^−1^). After incubation at 37°C for 30 min, a thin layer of methylcellulose hydrogel, which prevents cell adhesion and thus promotes cell spheroid assembly, formed in the bottom of the wells. Finally, SCs were trypsinized and inoculated into hydrogel‐coated plates at a density of 5000 cells in 150 μl medium per well and cultured for 24 h. The morphology of the assembled spheroids was observed under a phase‐contrast microscope, and their diameters were determined using ImageJ software (National Institute of Health, Bethesda, MD, USA).

For immunofluorescence staining, the SC spheroids were collected and fixed with 4% paraformaldehyde (Sigma‐Aldrich) for 30 min followed by permeabilization with 0.1% Triton X‐100 (Sigma‐Aldrich) and blocking with 5% goat serum (Vector Laboratories; Newark, CA, USA). The SC spheroids were then incubated overnight with antibodies against S100β, SOX10, laminin (Abcam; Cambridge, MA, USA), collagen I, fibronectin, BDNF, or HGF (GeneTex; Hsinchu, Taiwan). The next day, secondary antibodies conjugated to Alexa Fluor 546 or Alexa Fluor 633 (Thermo Fisher Scientific) were utilized to detect the primary antibodies. Finally, the samples were counterstained with 4′,6‐diamidino‐2‐phenylindole (DAPI), cleared with RapiClear 1.47 (SUNJin Lab; Hsinchu, Taiwan), and mounted before visualization with a confocal microscope (Carl Zeiss; Oberkochen, Germany).

For immunoblotting, SC spheroids were first lysed with RIPA buffer (Bio Basic; Amherst, NY, USA) supplemented with a protease inhibitor cocktail (Sigma‐Aldrich). After incubation at 95°C for 10 min to denature the proteins, sodium dodecyl sulfate–polyacrylamide gel electrophoresis on a 12% acrylamide gel (Bio‐Rad Laboratories, Hercules, CA) was performed at 145 V to resolve the samples. The proteins were then transferred to a polyvinylidene difluoride membrane (Cytiva; Marlborough, MA) at 200 mA for 2 h. The membrane was treated with 5% skim milk for 1 h and incubated with antibodies against c‐Jun, phosphorylated c‐Jun (Ser 63), MPZ (GeneTex), or β‐actin (Abcam) overnight at 4°C. The next day, horseradish peroxidase‐conjugated secondary antibodies (GeneTex) were utilized to probe the membrane. The signals were detected by using Amersham ECL Select Western Blotting Detection Reagent (Cytiva).

For qPCR, SC spheroids were lysed using TOOLSmart RNA Extractor (BioTools; New Taipei City, Taiwan) for the extraction of total RNA, which was then reverse‐transcribed into cDNA using a High‐Capacity cDNA Reverse Transcription Kit (Thermo Fisher Scientific) according to the manufacturer's manual. qPCR was carried out to determine the relative gene expression of samples using Genious 2X SYBR Green Fast qPCR Mix (ABclonal, Woburn, MA, USA) on a StepOnePlus Real‐Time PCR System (Thermo Fisher Scientific). The following primers were used: *Bdnf*, forward, 5′‐CTT GGA GAA GGA AAC CGC CT‐3′ and reverse, 5′‐GTC CAC ACA AAG CTC TCG GA‐3′; *Egr1*, forward, 5′‐CAG GAG TGA TGA ACG CAA GA‐3′ and reverse, 5′‐AGC CCG GAG AGG AGT AAG AG‐3′; *Egr2*, forward, 5′‐CCC TCT CCA AAA ACG GCT TC‐3′ and reverse, 5′‐CTG GGA TTT TGT CTA CGG CCT‐3′; *Gapdh*, forward, 5′‐GGG TGT GAA CCA CGA GAA AT‐3′ and reverse, 5′‐ACT GTG GTC ATG AGC CCT TC‐3′; *Gdnf*, forward, 5′‐CTG ACC AGT GAC TCC AAT ATG C‐3′ and reverse, 5′‐GCC TCT GCG ACC TTT CCC‐3′; *Hgf*, forward, 5′‐TCA TTG GTA AAG GAG GCA GCT ATA‐3′ and reverse, 5′‐CTG GCA TTT GAT GCC ACT CTTA‐3′; *Ngf*, forward, 5′‐TCA ACA GGA CTC ACA GGA GCA‐3′ and reverse, 5′‐GGT CTT ATC TCC AAC CCA CAC AC‐3′.

### 5.4 Preparation and characterization of SC spheroid‐derived CM

SCs that were assembled as spheroids or those in a single‐cell suspension were plated into six‐well culture plates at a density of 2 × 10^5^ cells per well followed by 48 h of incubation. The prepared CM was then collected and stored at −80°C before further use. To characterize the harvested CM, a rat cytokine antibody array (ab133992; Abcam) was utilized to measure 34 cytokines simultaneously via a chemiluminescence‐based method according to the manufacturer's instructions. The signal intensity of each cytokine was determined using ImageJ software and normalized to that of the positive control on each membrane.

### 5.5 Neurite outgrowth assay

DRG neurons were allowed to attach to Matrigel‐coated μ‐Slide 8 Wells overnight. The next day, the culture medium was replaced with SC‐derived CM and incubated for 48 h. To measure the growing neurites, cells were fixed and immunostained with an antibody against βIII tubulin (GeneTex) followed by treatment with Alexa Fluor 546‐conjugated secondary antibody. The neurites were visualized with a fluorescence microscope (Carl Zeiss), and five images were taken from randomly chosen fields within each sample. Next, ImageJ software plug‐in NeuronJ software was utilized to determine the average length of the longest neurite in each sample.^57^

### 5.6 SC proliferation and wound healing assay

Eight thousand SCs were inoculated into each well of 96‐well culture plates. The next day, the medium was replaced with SC‐derived CM and cultured for 48 h. Live/dead staining was performed by Cell‐Check Cell Viability/Cytotoxicity Assay (ABP Biosciences; Beltsville, MD, USA). Furthermore, a Cell Counting Kit‐8 assay (IMT Formosa New Materials, Kaohsiung, Taiwan)^75^ was performed to determine the viability of the treated SCs.

For the wound healing assay, 10,000 SCs were seeded into each well of 24‐well culture plates. The next day, a scratch wound was created in each cell monolayer using a pipette tip,^21^, ^76^, ^77^ and the culture medium was replaced with SC‐derived CM. The gap in cells created by the scratch was photographed with a phase‐contrast microscope, and the area of the cell‐free wound was measured using ImageJ software and normalized to the area of the gap immediately after wound creation.

### 5.7 Macrophage Transwell migration assay

Thirty thousand macrophages were suspended in 200 μl culture medium and added into a Transwell insert (8‐μm pore size; Jet Bio‐Filtration; Guangzhou, China). The well of the plate was supplemented with unconditioned SC medium or CM derived from SC suspensions or spheroids. After 24 h of incubation, the cells were fixed with 4% paraformaldehyde for 15 min and stained with 0.1% crystal violet (Sigma‐Aldrich) for 30 min. Finally, the cells remaining at the upper surface of the insert membrane were removed by a cotton swab. The stained cells were observed and photographed with a phase‐contrast microscope, and the number of cells was counted to determine the cell density.

### 5.8 Animal study

Rats were anesthetized by inhalation of 2–4% isoflurane, and carprofen (5 mg kg^−1^) was administered subcutaneously for pain control. After disinfecting the surgical site, an incision was made over the right thigh, and the underlying muscle was separated via blunt dissection. The exposed sciatic nerve was transected 5 mm proximal to its trifurcation and immediately reconstructed by end‐to‐end coaptation using a 9–0 nylon suture under a surgical microscope. Animals were allocated randomly to receive different treatments using a list randomizer (www.random.org/list/).^78^, ^79^ Immediately after reconstruction surgery, a total of 5 × 10^5^ SCs in spheroids (100 spheroids) or in single‐cell suspension were suspended in 10 μl of normal saline and loaded into a Hamilton syringe with a 24‐gauge needle. The needle was carefully inserted through the epineurium at both the upstream and downstream locations of the nerve coaptation site and guided along the nerve's axis, allowing for a controlled insertion depth of approximately 5 mm. Finally, the cells were administered slowly, and the syringe was held in place for 30 s before being removed.

To detect the injected cells, the sciatic nerve was harvested at 1 or 7 days postoperation, fixed in 10% phosphate‐buffered formalin for 24 h, and embedded in agarose before being sliced into 150‐μm‐thick sections with a vibratome (Leica Biosystems, Nussloch, Germany). After permeabilization with 2% Triton X‐100 for 10 min, the samples were stained with the lipophilic dye DiD (20 μg ml^−1^; Thermo Fisher Scientific) for 3 h followed by treatment with a mixture of the nucleic acid staining agent SYTO‐16 (5 mM; Thermo Fisher Scientific) and tissue‐clearing reagent (JelloX Biotech; Hsinchu, Taiwan) for 1 h. Images were acquired using a confocal microscope with a 40× air objective lens (Olympus, Tokyo, Japan). Alternatively, the collected nerve was fixed in 4% paraformaldehyde for 2 h, cryoprotected in 30% sucrose, embedded in OCT compound and sectioned at 10 μm.^80^ The sections were stained with primary antibodies against βIII tubulin, S100β, or CD68 (Abcam) and incubated at 4°C overnight followed by treatment with fluorescence‐conjugated secondary antibodies. After counterstaining with DAPI and mounting, the sections were observed under a confocal microscope.

Animal gait was evaluated using a GaitLab system (Viewpoint Behavior Technology; Civrieux, Rhone‐Alpes, France), an automatic turnkey system for gait analysis,^81^ before induction of sciatic nerve injury and 3 months after cell transplantation. Gait parameters, including stride length, paw contact pressure intensity, stance time, and duty factor, were assessed. Electrophysiological assessment was performed with an integrated sensor and data recorder (IX‐TA‐220; iWorx; Dover, NH, USA) 3 months after nerve repair. The sciatic nerve of the anesthetized animals was exposed and stimulated with a bipolar electrode (3 mA for 0.1 ms) 10 mm proximal to the transection site, and the generated CMAPs and time to deflection (latency) were recorded with a bipolar receiver electrode inserted into the gastrocnemius muscle.^82^ Furthermore, a force transducer (FT‐302; iWorx) was employed to measure the isometric contraction force exerted by the gastrocnemius and soleus muscles upon stimulation.

After electrophysiological evaluation, the gastrocnemius muscle of the affected limb was harvested, weighed, and normalized to that of the contralateral limb. The extent of muscle atrophy was defined as the proportion of muscle weight loss. In addition, the sciatic nerves were collected, fixed with 4% paraformaldehyde followed by 3% glutaraldehyde in 0.1 M cacodylate buffer and postfixed with 1% osmium tetroxide. Ultrathin sections were cut 20 mm from the injury site, stained with uranyl acetate and lead citrate, and observed by TEM (Hitachi; Tokyo, Japan). To determine the diameter of myelinated nerve fibers and the thickness of myelin sheaths, 10 images were taken from randomly chosen fields within each sample. Next, ImageJ software was utilized to analyze five nerve fibers within each of these images.

### 5.9 Statistical analysis

GraphPad Prism software (version 9.5.1; San Diego, CA, USA) was utilized for statistical analysis. For comparisons between two groups, Student's *t* test was performed. For comparisons among three or more groups, one‐way ANOVA followed by Tukey's correction was performed. All results presented in this article are expressed as the mean ± standard deviation. Differences with a *p* value <.05 were considered statistically significant. To determine the sample sizes for animal study in each group, G*Power software (Heinrich‐Heine‐Universität Düsseldorf, Düsseldorf, Germany) was utilized to carry out an a priori power analysis with an *α* error of .05 and power of .95.

## AUTHOR CONTRIBUTIONS

**Shih‐Shien Chen:** Conceptualization (equal); funding acquisition (equal); investigation (equal); methodology (equal); writing – original draft (equal). **Hsin‐Wen Wang:** Conceptualization (equal); investigation (equal); methodology (equal); validation (equal); writing – original draft (equal). **Pei‐Ching Yang:** Investigation (equal); methodology (equal); validation (equal). **Shih‐Hsien Chen:** Investigation (equal); validation (equal). **Chia‐Hsin Ho:** Investigation (equal); validation (equal). **Pei‐Ching Yang:** Investigation (equal); validation (equal). **Ying‐Chi Kao:** Investigation (equal); validation (equal). **Shao‐Wen Liu:** Investigation (equal); validation (equal). **Han Chiu:** Investigation (supporting); methodology (supporting). **Yu‐Jie Lin:** Investigation (supporting). **Er‐Yuan Chuang:** Methodology (supporting); resources (supporting). **Jen‐Huang Huang:** Methodology (supporting); resources (supporting). **Huang‐Kai Kao:** Methodology (supporting); resources (supporting). **Chieh‐Cheng Huang:** Conceptualization (equal); funding acquisition (equal); project administration (lead); resources (lead); writing – review and editing (lead).

## CONFLICT OF INTEREST STATEMENT

The authors declare no conflict of interests.

### PEER REVIEW

The peer review history for this article is available at https://www.webofscience.com/api/gateway/wos/peer-review/10.1002/btm2.10635.

## Supporting information


**FIGURE S1.** Relative mRNA levels of myelination markers *Egr1* and *Egr2* in SCs in a single‐cell suspension or spheroid configuration (n = 4). The data are presented as the mean ± SD. All *p* values were calculated by two‐tailed Student's *t* test. ***p < 0.005; ns, not significant.

**SUPPLEMENTARY VIDEO 1.** Three‐dimensional rendering displaying the distribution of transplanted Schwann cells (green), delivered as single cell suspensions, within the rat sciatic nerve (red) at seven days post‐operation.

**SUPPLEMENTARY VIDEO 2.** Three‐dimensional rendering illustrating the distribution of transplanted Schwann cells (green), delivered as cell spheroids, within the rat sciatic nerve (red) at seven days post‐operation.

## Data Availability

The data that support the findings of this study are available from the corresponding author upon reasonable request.
