# Supplementary material for: Schwann cells acquire a repair phenotype after assembling into spheroids and show enhanced in vivo therapeutic potential for promoting peripheral nerve repair
Source: Bioeng Transl Med. 2023 Dec 26;9(2):e10635. doi: 10.1002/btm2.10635 (PMC10905550; doi:10.1002/btm2.10635)
Supplement: Supplementary file 1 — FIGURE S1. Relative mRNA levels of myelination markers Egr1 and Egr2 in SCs in a single‐cell suspension or spheroid configuration (n = 4). The data are presented as the mean ± SD. All p values were calculated by two‐tailed Student's t test. ***p < 0.005; ns, not significant. [file BTM2-9-e10635-s002.pdf]

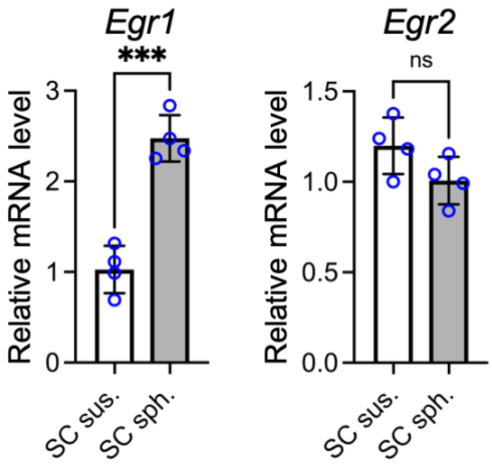

Figure S1. Relative mRNA levels of myelination markers *Egr1* and *Egr2* in SCs in a single-cell suspension or spheroid configuration (n = 4). The data are presented as the mean ± SD. All p values were calculated by two-tailed Student's t test. ***$p < 0.005$; ns, not significant.
